# Physical Activity and Beverage Consumption among Adolescents

**DOI:** 10.3390/nu8070389

**Published:** 2016-06-23

**Authors:** Maria del Mar Bibiloni, Asli Emine Özen, Antoni Pons, Marcela González-Gross, Josep A. Tur

**Affiliations:** 1 Research Group on Community Nutrition and Oxidative Stress, University of Balearic Islands, Palma de Mallorca E-07122, Spain; mar.bibiloni@uib.es (M.M.B.); antonipons@uib.es (A.P.); 2 CIBEROBN (Physiopathology of Obesity and Nutrition), Instituto de Salud Carlos III, Madrid E-28029, Spain; marcela.gonzalez.gross@upm.es; 3 Department of Gastronomy and Culinary Arts, Reha Midilli Foça Faculty of Tourism, Dokuz Eylül University, Foça-Izmir 35680, Turkey; asli.ozen@deu.edu.tr; 4 ImFINE Research Group, Department of Health and Human Performance, Faculty Physical Activity & Sport Sciences-INEF, Technical University of Madrid, Madrid E-28040, Spain

**Keywords:** beverage consumption, physical activity, adolescents

## Abstract

This study assessed the relationship between physical activity and beverage consumption among adolescents with a population based cross-sectional survey was carried out in the Balearic Islands, Spain (*n* = 1988; 12–17 years old). Body composition, educational and income level, physical activity (PA), and beverage consumption and energy intake were assessed. Sixty-two percent of adolescents engaged in >300 min/week of PA. Boys were more active than girls, younger adolescents were more active than older counterparts, low parental income was associated with physical inactivity, and time spent watching TV (including, TV, Internet or handheld cellular devices) was inversely associated with PA practice. The average beverage intake of the studied adolescents was 0.9 L/day, higher in boys than in girls. Beverage intake was positively associated with PA practice, and the highest amount of energy intake from beverages was observed in active boys and girls. Most of the studied adolescent population met the PA recommendations. Gender, age, parental income, and time spent watching TV were significant determinants of PA. Type and amount of beverages drunk varied according to gender and PA, and general daily total beverage intake was lower than recommended adequate fluid intake. PA behavior should be considered when analyzing beverage consumption in adolescents.

## 1. Introduction

Physical activity (PA) is important for a healthy development [1], and adequate levels of PA are currently included in most, if not all, public health guidelines for children and adolescents [2]. As PA increases cell metabolism, it causes an increase in body temperature, and sweating is the main way of maintaining heat balance during PA, especially in hot climates [2,3,4], increasing the needs of body water to keep adequate thermoregulation functioning, and even though fluid needs will be obviously different in both PA for health and PA as a sport, both show a common physiological basis. Contrary to former statements, current research indicates that young people have similar thermoregulatory ability to adults and that an adequate hydration contributes to a better thermoregulatory ability [5].

Insufficient voluntary fluid intake is common among active young people [6] and if they fail to replace fluid loss during and after exercise, it could lead to more heat storage in the body [7,8]. Hypohydration affects prolonged aerobic exercise more than it affects short, high-intensity anaerobic exercise [9,10]. Adolescents need to consume enough fluid to maintain an appropriate euhydration [7]. Fluid ingested before, during, and after exercise decreases dehydration, core temperature for a given heat production, heart rate, and cardiac strain [11], and then contributes to maintain skin blood flow and to increase exercise performance [12,13].

Beverages are an important source of fluids to maintain appropriate hydration level, but fluid requirements of physically active people are influenced by climate, age, sex, body size, sweat production, and food habits, as well as intensity and duration of PA [14,15,16]. One study has published beverage consumption in European adolescents [17], but most publications are mainly focused on sugar-sweetened beverages and overweight [18,19].

Therefore, fluid intake is essential for health, but also for a good PA performance, mainly in developing bodies, as in adolescents. However, scarce studies have assessed the relation between physical activity and beverage consumption among adolescents, and then recommendations do not distinguish between physically active and inactive subjects [20]. This study assessed the relationship between physical activity and beverage consumption among an adolescent population.

## 2. Materials and Methods

### 2.1. Study Design

The study is a population-based cross-sectional survey carried out in the Balearic Islands (Spain), a Mediterranean region, between 2007 and 2008.

### 2.2. Study Population

A multicenter study was performed on Balearic Islands’ adolescents aged 12–17 years. The population was selected by means of a multiple-step, simple random sampling, taking into account first the location of all the Balearic Islands (Palma de Mallorca 400,578 habitants, Calvià 50,328, Inca 30,651, Manacor 40,170, Maó 28,006, Eivissa 49,975, Llucmajor 34,618, Santa Margalida 11,672, S’Arenal 16,719, and Sant Jordi de Ses Salines 8048) and then by random assignment of the schools within each city. Sample size was stratified by age and gender. The socio-economic variable was considered to be associated to geographical location and type of school. As the selection of schools was done by random selection and fulfilling quota, this variable was also considered to be randomly assigned.

In order to calculate a representative number of adolescents, the variable Body Mass Index (BMI) with the greatest variance for this age group from the data published in the literature at the time the study was selected. The sampling was determined for the distribution of this variable; the confidence interval (CI) was established at 95% with an error ±0.25. The total number of subjects was uniformly distributed in the cities and proportionally distributed by sex and age. Exclusion criteria were: self-reported type 2 diabetes, pregnancy, alcohol or drug abuse, and non-directly related nutritional medical conditions.

The sample was oversized to prevent loss of information and as necessary to do the fieldwork in complete classrooms. In each school, classrooms were randomly selected among those of the same grade or level, and all the adolescents of one classroom were proposed to participate in the survey. A letter about the nature and purpose of the study informed parents or legal guardians. After receiving their written consent, the adolescents were considered for inclusion in the study. All responses of questionnaires were filled in by adolescents. After finishing the field study, the adolescents who did not fulfill the inclusion criteria were excluded. Finally, the sample was adjusted by a weight factor in order to balance the sample in accordance to the distribution of the Balearic Islands’ population and to guarantee the representativeness of each of the groups, already defined by the previously mentioned factors (age and sex). The final number of subjects included in the study was 1988 adolescents (82.8% participation), a representative sample of the Balearic Islands’ adolescent population. The reasons to not participate were: (a) the subject declined to be interviewed; and (b) the parents did not authorize the interview.

This study was conducted according to the guidelines laid down in the Declaration of Helsinki, and all procedures involving human subjects were approved by the Balearic Islands’ Ethics Committee (Palma de Mallorca, Spain) No. IB-530/05-PI.

### 2.3. General Questionnaire

Educational and income level of the parents was determined by means of a questionnaire incorporating the following questions: father’s and mother’s educational level (grouped according to years and type of education into low, <6 years at school; medium, 6–12 years of education; and high, >12 years of education), father’s and mother’s income (based on the occupation and classified as low, <12,000 euros/year; medium, 12,000–22,500 euros/year; and high, >22,500 euros/year), according to the methodology described by the Spanish Society of Epidemiology [21].

Information about smoking habits and alcohol intake was collected as described: smoking habit no; yes; occasionally, less than 1 cigarette/day; alcohol consumption no; frequently; occasionally, less than 1 drink/week.

### 2.4. Body Composition

Height was determined to the nearest millimeter using a mobile anthropometer (Kawe 44444, Kirchner & Wilhelm GmBH Co., KG, Asperg, Germany) with the subject’s head in the Frankfurt plane. Body weight was determined to the nearest 100 g using a digital scale (Tefal, sc9210, Groupe SEB, Rumilly, France). The subjects were weighed barefoot wearing light underwear, as previously described [22]. BMI was computed as weight (kg) per height squared (m^2^), and study participants were age- and gender-specific categorized using the BMI cut-offs developed and proposed by the International Obesity Task Force [23] and Cole et al. [24] definitions, and categorized as underweight (≤5th percentile), normal-weight (>5th–≤85th percentile), overweight (>85th percentile) and obese (≥95th percentile).

### 2.5. Physical Activity Assessment

Physical activity was assessed according to the guidelines for data processing and analysis of the short-form International Physical Activity Questionnaire [25], and its specific modification for adolescents (IPAQ-A) [26]. The specific types of activity assessed were: walking, moderate-intensity activities (i.e., PA at school) and vigorous-intensity activities (i.e., sport practice). An additional question about the time spent on a typical day sitting and watching TV (including, TV, Internet or handheld cellular devices), playing computer games, or talking with friends, but also time spent sitting at school and for homework, was used as an indicator variable of time spent at sedentary activities. According to the Patient-centered Assessment & Counseling for Exercise (PACE) + Adolescent Physical Activity Measure and existing guidelines [27,28], adolescents were also asked about the number of days with PA of at least 60 min/day of moderate-vigorous physical activity (≥3 Metabolic Equivalents or METs) during the past 7 days and during a typical week. The number of active days during the past week and during a typical week was averaged. On the basis of their total weekly PA (at least 60 min/day of moderate-vigorous physical activity on at least 5 day/week), the subjects were divided into 2 groups: inactive (<300 min/week) and active (≥300 min/week), according to the current PA recommendations for young people [28,29].

### 2.6. Assessment of Beverage Consumption and Energy Intake

Beverage consumption and energy intake were assessed using two non-consecutive 24 h diet recalls period, one was administered in the warm season (May–September) and another in the cold season (November–March) to account for the effect of seasonal variations. To bias brought on by day-to-day intake variability, the recalls were administered homogeneously from Monday to Sunday. Well-trained dieticians administered the recalls and verified and quantified the food records. Recalls were performed face-to-face at the participants’ classroom. Volumes and portion sizes were reported in natural units, household measures or with the aid of a manual of sets of photographs [30] Well-trained dieticians administered the recalls and verified and quantified the information obtained from the 24 h recalls.

Beverages were categorized into eleven groups: water (tap water, bottled water, and sparkling water), low-fat milk (low-fat and skimmed milk), whole-fat milk, diet soda (low calorie carbonated soft drinks), coffee/tea (coffee, black tea and herbal tea), fruit juice 100% (all kinds of natural fruit juice), non-diet soda (all kinds of carbonated sugared soft drinks), fruit juice (all kinds of fruit juice sweetened with sugar), alcohol (wine, beer, vodka, and whisky), energy/sports beverages, and others (carrot juice, beer without alcohol, chocolate milkshake, vanilla milkshake, strawberry milkshake, diet milkshake, soy milk, rice milk, oat milk, fermented milk drink with sugar, fermented milk drink, kefir, horchata, and sugar added iced tea).

Total energy intake (TEI) from whole diet and from beverages were calculated using a computer program (ALIMENTA^®^, NUCOX, Palma, Spain) based on Spanish [31,32] and European Food Composition Tables [33], and complemented with food composition data available for Balearic food items [34]. Identification of misreporters: an energy intake (EI)/basal metabolic rate (BMR) ratio <0.92 (boys) and <0.85 (girls) was considered to represent under-reporters [35], and an EI/BMR ≥ 2.4 as over-reporters [36].

### 2.7. Statistical Analyses

Statistical Package for the Social Sciences for Windows version 21.0 (SPSS Inc., Chicago, IL, USA) was used. Absolute numbers and percentages of participants according to physical activity practice were calculated by using a general lineal model adjusted by age, sex, and BMI. Significant differences in mean daily beverage and energy intake were tested by means of ANOVA. Significant differences in percentages were tested by means of *χ*^2^. Crude and adjusted by potential confounders (age and sex), odds ratios (OR) and 95% confidence intervals (CI) were calculated to examine the relationship between the risk of being inactive and socio-demographic and lifestyle characteristics. Linear regression analysis was used to evaluate associations between physical activity and beverage consumption. For all statistical tests, *p* < 0.05 was taken as the significant level.

## 3. Results

### 3.1. Socio-Demographic and Lifestyle Characteristics of the Population

Twenty-two percent of the final sample did not report their energy intake accurately (underreporters 20% and overreporters 2%) and were excluded from further analysis. Table 1 shows the socio-demographic and lifestyle characteristics of the study population according to PA level. Sixty-two percent of the adolescents met the recommendations (>300 min/week). Sex, age, parental education level and income, alcohol intake and time spent watching TV, were significant determinants of PA practice. Boys and younger adolescents had lower risk of being inactive. Adolescents whose father had lower income were more likely to be inactive. The length of time spent watching TV was positively associated with PA, and the risk of being inactive was lower among adolescents who watched TV ≤ 1 h/day.

### 3.2. Daily Beverage Consumption and Energy Intake

Daily beverage, energy intake and percentage of consumers among adolescents related with PA practice are shown in Table 2. Beverage and energy intake were obtained only from those adolescents who consumed the drinks. Physically active girls had higher mean daily water, total beverage, and beverage TEI, and lower fruit drink intake than inactive girls. Physically active boys showed higher total beverage, dietary TEI, and beverage TEI than inactive boys. Gender differences were also observed: inactive boys showed higher consumption of whole-fat milk, and soda, as well as higher dietary and beverage TEI than inactive girls; and active boys showed higher consumption of whole milk, fruit drinks, and soda consumption, and dietary and beverage TEI, as well as lower low-fat milk than active girls. Percentage of consumers of whole milk, fruit juice and fruit drinks, and energy/sport beverages are higher among active boys; proportion of consumers of low-fat and whole milk, fruit juice, and energy/sport beverages are higher among active girls. Among inactive adolescents, boys showed higher percentage of consumers of low-fat milk, diet soda, and other beverages, whereas girls showed higher proportion of consumers of fruit drinks, soda, coffee/tea, and other beverages than active peers.

### 3.3. Beverage Consumption According to Seasons

Table 3 and Table 4 (boys and girls, respectively) show daily beverage, energy intake and percentage of consumers among active and inactive adolescents according to seasons. More water, diet soda, and total beverages are consumed in the warm than in the cold season in both active and inactive boys and girls. Alcoholic beverages as well as dietary and beverage total energy intake are more consumed in the cold season. In boys, activity in the cold season increased water, fruit juice (100%) and dietary TEI and decreased coffee/tea consumption, whereas in the warm season there were increased water, low-fat milk and total beverage consumption, and decreased whole milk, fruit juice (100%), fruit drinks, coffee/tea and alcoholic beverage consumption. In girls, activity in the cold season increased diet soda consumption, whereas in the warm season there were increased water, fruit juice (100%), diet soda, energy/sport beverages, and total beverage consumption.

### 3.4. Beverage Consumption According to Physical Activity

**Figure 1:**
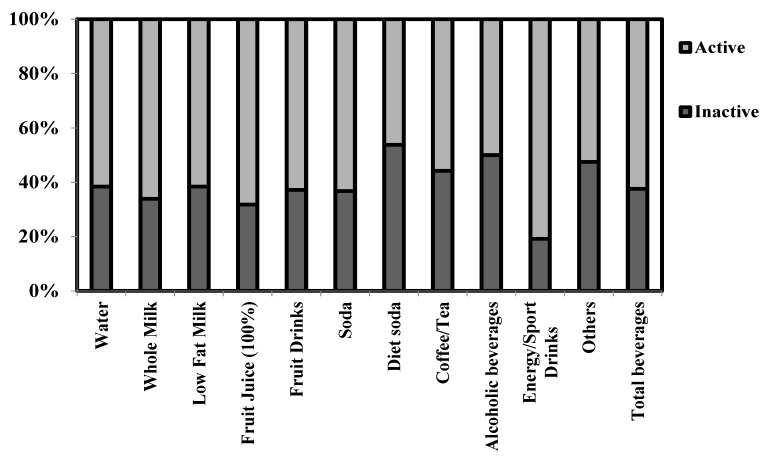
Proportions of consumers of each beverage according to physical activity level (others include carrot juice, beer without alcohol, chocolate milkshake, vanilla milkshake, strawberry milkshake, diet milkshake, soy milk, rice milk, oat milk, fermented milk drink with sugar, fermented milk drink, kefir, horchata, and sugar added iced tea).

Proportions of consumers of each beverage according to physical activity level are shown in Figure 1. More than half of the consumers for each beverage were physically active. Highest consumption of energy/sport beverage drinkers was found among active adolescents, whereas the highest proportion of diet soda drinkers was observed among inactive subjects.

Results of the linear regression analysis on the association between PA level and beverage consumption are shown in Table 5. A statistically significant and positive association was observed between PA level and total beverage consumption in model 2 (adjusted data by age, gender, total energy intake and BMI; *p* = 0.032).

## 4. Discussion

Fluid intake either from water, beverages or foods is necessary for physical and mental function [5,14]. However, fluid needs for individuals are variable depending on age, body size, PA level, perspiration, food habits, and environmental conditions [14,15,16]. Hydration plays an important role in the ability to perform PA, but not in short bout exercise (power or anaerobic activities) [37]. Physical performance is impaired by dehydration, even during relatively short-duration, intermittent exercise, which may provoke changes in cardiovascular, thermoregulatory, metabolic, and central nervous function that become greater as dehydration worsens [38]. Therefore, hydration status should be controlled among people practicing PA, and beverages are an important source of fluids to maintain appropriate levels of hydration.

In addition to normal meals and fluid intake, appropriate prehydrating with beverages is useful to start the PA euhydrated, mainly taking water as the main hydration source. It should be started at least several hours before the activity. During exercise, consuming beverages will allow to prevent dehydration to avert compromised performance. After exercise, beverage consumption will allow to replace any fluid deficit [39].

Participating in regular PA and eating a balanced diet are recognized to be beneficial for health [40], but physical inactivity of adolescents has been usually reported [1,41,42]. In the present analysis most of the studied adolescents were physically active, which is similar to previous results reported in developed countries [43,44,45,46,47], and higher than in developing countries [48,49], measured by means of self-reported instruments of PA assessment similar to the method used in this study. In agreement with previous findings [1,41,42,43,44,45,46,47,48,49,50,51], boys were more active than girls, younger adolescents were physically more active than their older counterparts, low parental income was associated with increased likelihood of being physically inactive, and time spent watching TV was inversely associated with PA. Displacement of PA by TV viewing decreases energy expenditure [41]. In addition, during TV viewing the consumption of beverages, mainly sugar-sweetened beverages, may increase the total energy intake [19,50]. It has been shown among our inactive girls that showed higher total energy intake from beverages than active girls.

Consumption of beverages varied according to sex and PA. The average beverage intake of the studied adolescents was 0.9 L/day, higher in boys than in girls, but these values were lower than the European Food Safety Authority [20] recommended adequate fluid intake (9–13 years: boys 2.1 L/day and girls 1.9 L/day; ≥14 years: boys 2.5 L/day and girls 2.0 L/day), and lower than mean beverage consumption of European adolescents (1.6 L/day in boys and 1.3 L/day in girls) [16]. However, these recommendations of adequate intakes are applied only to conditions of moderate environmental temperature and moderate PA levels.

Accordingly, water, soft drinks and total beverage consumption were higher in the warm than the cold season, whereas alcohol and beverage total energy intake were higher in the cold season. These results are quite different from previous results in Korean school students that showed no differences between winter and summer on amounts of beverage per day and the daily energy intake from beverage consumption [52]. However, Korean girls, similarly to ours, also showed higher consumption of sweetened beverages in the warm season. Furthermore, no evidence that fluid consumption among children was significantly related to the mean temperature in modern conditions [53]. The 1997 Spanish National Household Health Survey showed that Spanish adolescents did not show seasonality of alcohol drinking habits, and they were only weekly drinkers, mainly on weekends [54]. Then, it could be inferred that most consumed beverages to recover fluid loss (i.e., water) are linked to the environmental heat, whereas consumption of hot (i.e., coffee or tea) and alcoholic beverages, with an additional calorific action, are linked to the environmental cold. However, changes in beverage consumption are mainly related to PA practice, and when these differences appeared in seasons, they may be the result of both PA and season, according to loss or need of fluids and nutrients.

As expected, total beverage intake of the studied adolescents was positively associated with PA. The highest amount of beverage TEI was observed in active boys and girls, in spite that it was lower than beverage TEI of European adolescents [16]. Active boys preferred to consume high energy beverages such as whole fat milk, or fruit drinks, whereas active girls preferred to consume low energy beverage such as low-fat milk, which may be related to the girls’ preference for a slim body shape [55].

Except diet soda and alcoholic beverage drinkers, more than 50% of all other beverage consumers met the PA recommendations. It is usually recommended to increase the daily PA level to lose weight in conjunction with better diet quality may contribute to better body weight control and prevention of various chronic diseases [56], but PA is also important for disease prevention, weight maintenance and overall health, including optimal growth and development for children and adolescents, and then it is usually recommended both to control dietary intake and also to practice PA. In this way, the highest proportion of diet soda drinkers was found among inactive adolescents. Perhaps adolescents drink diet soda with the recognition that they have not been expending enough energy.

Otherwise, exercise increases the requirement of many electrolytes due to their loss via sweat [57], and isotonic beverages are used for recovery of missing water and electrolytes during or after physical activity [57,58]. The highest proportion of energy/sport drink consumers was found among active adolescents, as previously registered [59], and this behavior might be due to compliment higher energy expenditure but also to imitate what done by elite sportsmen. The consumption of these beverages among adolescents may be recommended only after vigorous and prolonged activity [59].

### Strengths and Limitations of the Study

This is the first time that the consumption of any kind of beverage and their relationship with PA has been assessed among adolescents. This study also assessed the percentage of the population consuming the different beverages, avoiding the sometimes misleading mean intake data.

This study has also limitations. Dietary questionnaires have inherent limitations, mainly because they are subjective in nature. The difficulties to assess food and beverage intake in humans are well-known. However, in many cases, self-reporting is the only feasible method of assessing dietary intake in epidemiological studies. Using single 24-h dietary recalls is not the best method to represent typical consumption patterns of individuals, because food and beverage consumption individually vary from day to day and 24-h dietary recalls have limitations related to memory and bias [60]. PA was assessed according to self-reported questionnaire, and may be affected by recall bias because adolescents might not able to accurately remember and capture their activities [61].

## 5. Conclusions

Most of the studied adolescent population met the PA recommendations. Gender, age, parental income, and time watching TV were significant determinants of PA. Consumption of beverages varied according to gender and PA, and daily total beverage intake was lower than recommended adequate fluid intake. PA behavior should be considered when assessing beverage consumption in adolescents.

## Figures and Tables

**Table 1:** Socio-demographic and lifestyle characteristics, according to physical activity and multivariable analysis of risk factors for low physical activity versus moderate and high physical activity groups.

	Inactive ^1^	Active ^2^	*χ^2^*	Risk of Being Inactive
	*n* (%) ^3^	*n* (%) ^3^		OR ^4^ (95% CI)
**Sex**			<0.0001	
Male	171 (23.2)	568 (76.8)		0.31 (0.25–0.39) *
Female	415 (51.1)	397 (48.9)		1.00
**Age (Years)**			0.001	
12–13	112 (30.5)	256 (69.5)		0.59 (0.42–0.82) *
14–15	274 (38.5)	437 (61.5)		0.82 (0.63–1.06)
16–17	220 (46.6)	252 (53.4)		1.00
**Father’s Education Level ^5^**			<0.0001	
Low	200 (41.4)	283 (58.6)		0.94 (0.63–1.39)
Medium	257 (39.2)	397 (60.8)		1.08 (0.78–1.49)
High	124 (29.9)	290 (70.1)		1.00
**Mother’s Education Level ^5^**			<0.0001	
Low	188 (42.6)	254 (57.4)		1.48 (0.99–2.20)
Medium	270 (38.8)	426 (61.2)		1.27 (0.92–1.75)
High	126 (30.6)	287 (69.4)		1.00
**Father’s Income**			<0.0001	
Low	239 (42.6)	321 (57.4)		1.66 (1.12–2.45) *
Medium	252 (35.7)	452 (64.3)		1.28 (0.90–1.83)
High	87 (30.3)	200 (69.7)		1.00
**Mother’s Income**			0.013	
Low	245 (31.1)	544 (68.9)		0.72 (0.47–1.10)
Medium	206 (34.4)	393 (65.6)		0.68 (0.46–1.01)
High	60 (36.8)	103 (63.2)		1.00
**BMI (kg/m^2^)**			0.870	
Underweight	14 (41.5)	20 (58.5)		1.08 (0.50–2.30)
Normal weight	427 (37.8)	703 (62.2)		0.83 (0.56–1.23)
Overweight	99 (39.7)	148 (60.3)		0.94 (0.60–1.47)
Obese	55 (39.2)	85 (60.8)		1.00
**Smoking**			0.473	
Yes	40 (43.4)	52 (56.6)		1.09 (0.67–1.77)
Occasionally	149 (37.8)	245 (62.2)		1.01 (0.78–1.30)
No	400 (37.6)	665 (62.4)		1.00
**Alcohol Intake**			0.032	
Yes	336 (36.1)	595 (63.9)		1.02 (0.80–1.31)
No	254 (41.0)	366 (59.0)		1.00
**Time Spent Watching TV**			0.001	
<1 h/day	74 (31.0)	165 (69.0)		0.68 (0.48–0.97) *
1–2 h/day	328 (36.7)	565 (63.3)		0.82 (0.64–1.04)
>2 h/day	184 (43.9)	235 (56.1)		1.00

^1^ Physical activity < 300 min/week; ^2^ Physical activity ≥ 300 min/week; ^3^ Percentage of population was tested by *χ*^2^; ^4^ Odds ratios (ORs) and 95% Confidence Interval (95% CI) were adjusted by age and gender; * Odds ratios within a column, for a characteristic, were statistically significant from 1.00 (*p* < 0.05); ^5^ Educational level of parents: low: <6 years, medium 6–12 years, high: >12 years.

**Table 2:** Daily beverage (mL), energy intake and percentage of consumers among active and inactive adolescents.

Beverages (mL)	Boys					Girls				
	Inactive (*n* = 171)		Active (*n* = 568)		*p* Value ^1^	Inactive (*n* = 415)		Active (*n* = 397)		*p* Value ^1^
	Mean ± SEM	% Consumers	Mean ± SEM	% Consumers		Mean ± SEM	% Consumers	Mean ± SEM	% Consumers	
Water	760.9 ± 45.9	73.7	871.3 ± 28.7	73.1	0.058	728.0 ± 28.1	80.7	885.1 ± 33.5	81.9	0.001
Low-Fat Milk	281.4 ± 17.0	27.5	301.5 ± 10.2 *	25.2	0.323	272.3 ± 9.1	32.0	277.1 ± 8.7	37.0	0.701
Whole Milk	298.5 ± 20.3	55.6	301.9 ± 7.9 *	62.9	0.854	276.3 ± 9.9	44.3	265.0 ± 8.0	46.9	0.371
Fruit Juice (100%)	200.0 ± 20.7	4.7	246.3 ± 22.3	7.0	0.363	237.5 ± 26.1	7.7	213.5 ± 8.8	12.1	0.318
Fruit Drinks	370.8 ± 20.9	23.4	338.3 ± 15.9 **	34.3	0.466	312.6 ± 22.0	36.1	269.7 ± 12.4	32.0	0.107
Soda	441.6 ± 31.5	33.9	474.0 ± 20.4 ***	33.6	0.428	400.4 ± 19.7	26.5	365.4 ± 22.1	24.7	0.236
Diet Soda	330.0 ± 30.1	2.3	396.7 ± 36.9	0.5	0.586	266.7 ± 36.7	0.7	410.0 ± 49.3	0.8	0.159
Coffee/Tea	99.6 ± 18.8	7.0	85.0 ± 9.0 *	7.9	0.469	101.2 ± 12.1	10.8	131.3 ± 21.1	7.1	0.188
Alcoholic Beverages	220.0 ± 15.1	0.6	225.0 ± 20.5	0.4	0.983	250.0 ± 23.0	0.2	280.0 ± 35.0	0.3	0.323
Energy/Sport Beverages	495.0 ± 16.5	1.2	337.1 ± 29.8	2.5	0.115	233.3 ± 36.7	0.7	330.0 ± 26.4	1.8	0.133
Others ^2^	461.3 ± 46.1	19.3	531.2 ± 64.4	10.9	0.501	501.7 ± 61.2	22.7	573.8 ± 67.2	16.6	0.443
Total Beverage	1004.4 ± 46.5	100.0	1141.7 ± 27.9	100.0	0.015	1003.9 ± 29.0	100.0	1060.3 ± 30.8	100.0	0.012
Dietary TEI (kcal/day)	2254.4 ± 51.3 ***	100.0	2378.7 ± 32.8 ***	100.0	0.042	1952.6 ± 28.5	100.0	1923.8 ± 30.2	100.0	0.486
Beverage TEI (kcal/day)	253.9 ± 44.1 ***	100.0	274.4 ± 23.7 ***	100.0	0.006	238.3 ± 25.9	100.0	230.8 ± 31.4	100.0	0.002

Inactive: <300 min/week. Active: >300 min/week; ^1^ *p* value: Active vs. inactive boys, and active vs. inactive girls by ANOVA; Active boys vs. active girls, and inactive boys vs. inactive girls by ANOVA (* *p* < 0.05; ** *p* < 0.01; *** *p* < 0.001); TEI = total energy intake; ^2^ Others include carrot juice, beer without alcohol, chocolate milkshake, vanilla milkshake, strawberry milkshake, diet milkshake, soy milk, rice milk, oat milk, fermented milk drink with sugar, fermented milk drink, kefir, horchata, and sugar added iced tea.

**Table 3:** Daily beverage (mL), energy intake and percentage of consumers among active and inactive boys according to the season.

Beverages (mL)	Inactive Boys (*n* = 171)					Active Boys (*n* = 568)				
	Cold Season		Warm Season		*p* Value ^1^	Cold Season		Warm Season		*p* Value ^1^
	Mean ± SEM	% Consumers	Mean ± SEM	% Consumers		Mean ± SEM	% Consumers	Mean ± SEM	% Consumers	
Water	721.6 ± 51.4	65.3	855.4 ± 95.2	76.2	0.007	803.7 ± 30.9 **	63.0	1013.0 ± 59.4 **	78.0	0.001
Low-Fat Milk	275.9 ± 24.1	17.4	289.5 ± 23.0	36.5	0.700	277.5 ± 12.2	12.0	338.8 ± 16.9 *	30.0	0.003
Whole Milk	281.0 ± 14.7	67.2	368.4 ± 82.8	46.5	0.086	295.6 ± 8.9	66.0	324.6 ± 17.5 *	46.0	0.131
Fruit Juice (100%)	200.0 ± 27.2	3.1	280.6 ± 42.0	5.8	0.325	260.4 ± 35.5 *	1.0	225.0 ± 17.1 *	9.0	0.444
Fruit Drinks	313.3 ± 47.8	16.8	490.0 ± 58.3	26.0	0.018	314.0 ± 17.9	21.0	389.2 ± 30.9 *	36.0	0.026
Soda	400.0 ± 45.4	25.5	498.8 ± 40.4	37.7	0.400	459.3 ± 22.5	23.0	512.3 ± 44.4	38.0	0.245
Diet Soda	320.6 ± 27.1	1.9	421.2 ± 38.3	3.9	0.056	330.6 ± 25.3	0.3	430.0 ± 23.0	0.6	0.043
Coffee/Tea	94.5 ± 19.4	9.2	125.0 ± 75.0	5.8	0.370	88.5 ± 10.6 *	9.0	68.8 ± 13.1 *	4.0	0.409
Alcoholic Beverages	330.0 ± 18.1	1.0	170.0 ± 20.5	0.4	0.007	330.2 ± 22.5	0.6	120.6 ± 35.8 *	0.3	0.005
Energy/Sport Beverages	330.9 ± 25.8	0.5	377.8 ± 58.9	1.8	0.779	344.0 ± 33.4	4.0	346.7 ± 55.6	3.0	0.794
Others ^2^	485.0 ± 46.1	29.9	350.3 ± 70.0	9.8	0.002	480.2 ± 58.7	12.0	463.8 ± 63.5	8.0	0.097
Total Beverage	913.5 ± 47.5	100.0	1281.1 ± 51.2	100.0	0.001	1025.0 ± 29.1	100.0	1454.9 ± 60.5 *	100.0	0.001
Dietary TEI (kcal/day)	2373.2 ± 95.1	100.0	2213.8 ± 64.5	100.0	0.045	2511.6 ± 40.5 *	100.0	2230.3 ± 53.6	100.0	0.032
Beverage TEI (kcal/day)	324.1 ± 47.8	100.0	236.8 ± 22.7	100.0	0.009	341.3 ± 52.1	100.0	250.9 ± 19.9	100.0	0.007

Inactive: <300 min/week. Active: >300 min/week; ^1^ *p* value: Warm vs. cold season by ANOVA; Active vs. inactive by ANOVA (* *p* < 0.05; ** *p* < 0.01; *** *p* < 0.001); TEI = total energy intake; ^2^ Others include carrot juice, beer without alcohol, chocolate milkshake, vanilla milkshake, strawberry milkshake, diet milkshake, soy milk, rice milk, oat milk, fermented milk drink with sugar, fermented milk drink, kefir, horchata, and sugar added iced tea.

**Table 4:** Daily beverage (mL), energy intake and percentage of consumers among active and inactive girls according to the season.

Beverages (mL)	Inactive Girls (*n* = 415)					Active Girls (*n* = 397)				
	Cold Season		Warm Season		*p* Value ^1^	Cold Season		Warm Season		*p* Value ^1^
	Mean ± SEM	% Consumers	Mean ± SEM	% Consumers		Mean ± SEM	% Consumers	Mean ± SEM	% Consumers	
Water	705.8 ± 32.4	78.5	754.1 ± 54.1	83.1	0.003	838.8 ± 43.8	80.1	950.2 ± 51.6 *	83.8	0.006
Low-Fat Milk	273.8 ± 10.7	35.0	268.4 ± 17.3	32.1	0.590	266.3 ± 9.7	36.9	288.4 ± 14.6	39.1	0.206
Whole Milk	272.3 ± 11.6	46.2	280.1 ± 18.3	41.6	0.353	262.7 ± 7.9	48.4	272.1 ± 21.8	43.3	0.318
Fruit Juice (100%)	243.3 ± 53.3	4.2	233.5 ± 16.1	10.6	0.177	193.5 ± 6.5	7.0	282.0 ± 15.0 *	15.0	0.027
Fruit Drinks	331.9 ± 30.7	35.2	268.9 ± 17.1	38.8	0.188	273.8 ± 16.9	29.9	260.5 ± 13.5	33.4	0.223
Soda	403.5 ± 24.5	26.1	393.7 ± 33.2	22.9	0.419	358.9 ± 28.5	24.4	374.3 ± 35.1	24.6	0.534
Diet Soda	246.7 ± 56.7	0.8	266.7 ± 65.7	0.6	0.521	390.0 ± 39.3 *	0.6	430.0 ± 33.3 *	1.8	0.154
Coffee/Tea	106.8 ± 14.9	9.8	92.5 ± 20.4	10.5	0.450	152.4 ± 41.0	5.8	120.0 ± 50.0	5.4	0.550
Alcoholic Beverages	270.0 ± 33.0	0.4	243.2 ± 28.4	0.1	0.005	295.0 ± 33.0	0.2	274.2 ± 43.0	0.3	0.009
Energy/Sport Beverages	332.0 ± 36.5	0.3	185.0 ± 45.2	1.3	0.667	321.0 ± 25.8	0.9	337.0 ± 18.7 *	2.4	0.425
Others ^2^	526.2 ± 88.4	23.7	490.3 ± 43.5	21.3	0.361	569.3 ± 41.0	16.0	604.6 ± 34.3	17.4	0.141
Total Beverage	949.9 ± 34.6	100.0	1142.5 ± 50.8	100.0	0.007	925.7 ± 35.4	100.0	1320.2 ± 53.4 *	100.0	0.001
Dietary TEI (kcal/day)	1951.6 ± 35.6	100.0	1954.9 ± 46.9	100.0	0.356	1898.0 ± 36.7	100.0	1942.2 ± 52.8	100.0	0.242
Beverage TEI (kcal/day)	236.1 ± 24.9	100.0	238.5 ± 32.1	100.0	0.754	224.0 ± 15.1	100.0	228.7 ± 25.9	100.0	0.542

Inactive: <300 min/week. Active: >300 min/week; ^1^ *p* value: Warm vs. cold season by ANOVA; Active vs. inactive by ANOVA (* *p* < 0.05; ** *p* < 0.01; *** *p* < 0.001); TEI = total energy intake; ^2^ Others include carrot juice, beer without alcohol, chocolate milkshake, vanilla milkshake, strawberry milkshake, diet milkshake, soy milk, rice milk, oat milk, fermented milk drink with sugar, fermented milk drink, kefir, horchata, and sugar added iced tea.

**Table 5:** Association between physical activity and beverage consumption.

Beverages	Physical Activity					
	Model 1 ^1^			Model 2 ^2^		
	β	SE	*p*	β	SE	*p*
Water	0.164	0.130	0.205	0.190	0.137	0.166
Low Fat Milk	−0.021	0.029	0.478	−0.018	0.031	0.566
Whole Milk	0.018	0.037	0.620	0.046	0.038	0.213
Fruit Juice (100%)	0.016	0.014	0.246	0.021	0.015	0.157
Fruit Drinks	−0.023	0.040	0.564	−0.029	0.043	0.493
Soda	0.081	0.047	0.087	0.089	0.050	0.073
Diet Soda	0.011	0.007	0.092	0.010	0.007	0.152
Coffee/Tea	0.006	0.007	0.435	0.008	0.007	0.300
Alcoholic Beverages	0.002	0.005	0.674	0.002	0.006	0.725
Energy/Sport Beverages	0.003	0.009	0.772	0.003	0.010	0.743
Others ^3^	−0.010	0.020	0.609	−0.011	0.021	0.611
Total Beverage	0.265	0.147	0.072	0.313	0.146	0.032

^1^ Adjusted by age and gender; ^2^ Adjusted by age, gender, total energy intake and BMI; ^3^ Others include carrot juice, beer without alcohol, chocolate milkshake, vanilla milkshake, strawberry milkshake, diet milkshake, soy milk, rice milk, oat milk, fermented milk drink with sugar, fermented milk drink, kefir, horchata, and sugar added iced tea.
